# Phenolic Acid Investigation and In Vitro Antioxidant and Antiacetylcholinesterase Potentials of *Galeopsis* spp. (*Lamiaceae*) from Romanian Flora

**DOI:** 10.3390/ph18040599

**Published:** 2025-04-20

**Authors:** Roxana Maria Golu, Cornelia Bejenaru, Ludovic Everard Bejenaru, Adina-Elena Segneanu, Andrei Biţă, Antonia Radu, Adriana Cosmina Tîrnă, Maria Viorica Ciocîlteu, George Dan Mogoşanu, Johny Neamţu, Oana Elena Nicolaescu

**Affiliations:** 1 Doctoral School, University of Medicine and Pharmacy of Craiova, 2 Petru Rareş Street, 200349 Craiova, Romania; mavirofarm@yahoo.com (R.M.G.); tirna.adriana@gmail.com (A.C.T.); 2 Department of Pharmaceutical Botany, Faculty of Pharmacy, University of Medicine and Pharmacy of Craiova, 2 Petru Rareş Street, 200349 Craiova, Romania; cornelia.bejenaru@umfcv.ro (C.B.); antonia.radu@umfcv.ro (A.R.); 3 Drug Research Center, Faculty of Pharmacy, University of Medicine and Pharmacy of Craiova, 2 Petru Rareş Street, 200349 Craiova, Romania; andreibita@gmail.com (A.B.); maria.ciocilteu@umfcv.ro (M.V.C.); george.mogosanu@umfcv.ro (G.D.M.); johny.neamtu@umfcv.ro (J.N.); oana.nicolaescu@umfcv.ro (O.E.N.); 4 Department of Pharmacognosy & Phytotherapy, Faculty of Pharmacy, University of Medicine and Pharmacy of Craiova, 2 Petru Rareş Street, 200349 Craiova, Romania; 5 Institute for Advanced Environmental Research, West University of Timişoara (ICAM–WUT), 4 Oituz Street, 300086 Timişoara, Romania; adina.segneanu@e-uvt.ro; 6 Department of Instrumental and Analytical Chemistry, Faculty of Pharmacy, University of Medicine and Pharmacy of Craiova, 2 Petru Rareş Street, 200349 Craiova, Romania; 7 Department of Physics, Faculty of Pharmacy, University of Medicine and Pharmacy of Craiova, 2 Petru Rareş Street, 200349 Craiova, Romania; 8 Department of Pharmaceutical Technology, Faculty of Pharmacy, University of Medicine and Pharmacy of Craiova, 2 Petru Rareş Street, 200349 Craiova, Romania

**Keywords:** *Galeopsis* spp., *Lamiaceae*, Romanian flora, phenolic acids, UHPLC/UV/MS analysis, antioxidant activity, acetylcholinesterase inhibitory activity

## Abstract

**Background/Objectives** *Galeopsis* spp. (*Lamiaceae*) are widely distributed across extensive areas in Romania, being used mainly for their sedative, neuroprotective, antioxidant, anti-inflammatory, expectorant, astringent, and diuretic properties. The paper reports for the first time the investigation of the total phenolic content (TPC), total flavonoid content (TFC), and phenolic acid profile in the roots, aerial parts, and leaves from three wild-grown *Galeopsis* spp. (*G. bifida* Boenn., *G. speciosa* Mill., and *G. tetrahit* L.), along with their antioxidant and acetylcholinesterase (AChE) inhibitory potentials. **Methods**: The ultra-high-performance liquid chromatography/ultraviolet/mass spectrometry (HPLC/UV/MS) method was used for the identification and quantification of key phenolic acids. The spectrophotometric method was applied for the determination of TPC, TFC, 2,2-diphenyl-1-picrylhydrazyl (DPPH), and 2,2′-azino-*bis*(3-ethylbenzothiazoline-6-sulfonic acid) (ABTS) radical scavenging activities and also the ferric-reducing antioxidant power (FRAP). High-performance thin-layer chromatography (HPTLC) was employed for the assessment of in situ antioxidant (DPPH assay) and AChE inhibitory potentials. **Results**: *Galeopsis* spp. exhibit significant polyphenol accumulation. Chlorogenic acid was the most abundant compound, with the highest levels detected in *G. tetrahit* leaves (22,347.907 ± 1117.395 μg/g), followed by *G. tetrahit* aerial parts (11,678.509 ± 583.925 μg/g) and *G. speciosa* leaves (8712.628 ± 435.631 μg/g). *G. tetrahit* leaves had the highest DDPH radical scavenging activity, with a half-maximal inhibitory concentration (IC_50_) of 0.458 ± 0.03 mg/mL, demonstrating a markedly stronger antioxidant effect. Leaves consistently showed the strongest DPPH activity across all species, with *G. speciosa* leaves also displaying a low IC_50_ value of 0.789 ± 0.03 mg/mL, comparable to *G. tetrahit*. Aerial parts exhibited an intermediate effect, with *G. bifida* aerial parts showing an IC_50_ of 8.102 ± 0.49 mg/mL, while *G. tetrahit* aerial parts demonstrated stronger activity at 1.511 ± 0.11 mg/mL. AChE inhibition activity increased progressively from the roots to aerial parts to leaves, with leaves consistently exhibiting the strongest inhibitory effects across all *Galeopsis* spp. *G. tetrahit* leaves had the strongest inhibition, with an IC_50_ of 4.002 ± 0.32 mg/mL, followed by *G. speciosa* leaves (6.92 ± 0.14 mg/mL) and *G. bifida* leaves (6.97 ± 0.68 mg/mL). **Conclusions**: Our study provides a comprehensive analysis of the phenolic acid content, in vitro antioxidant activity, and neuroprotective potential of three *Galeopsis* spp. (*G. bifida*, *G. speciosa*, and *G. tetrahit*) from the southwestern Romanian flora.

## 1. Introduction

The genus *Galeopsis* (*Lamiaceae*) comprises annual herbaceous species distributed throughout Europe and Asia but naturalized in other regions of the world (North America) [1,2,3]. In Europe, nine *Galeopsis* spp. are documented, of which seven occur in Romania (*G. angustifolia* Ehrh., *G. bifida* Boenn., *G. ladanum* L., *G. pubescens* Besser, *G. segetum* Neck., *G. speciosa* Mill., and *G. tetrahit* L.) [1,2,4,5,6].

Plants of the genus *Galeopsis* feature four-sided, highly branched stems which, in certain species, may exhibit rigid, appressed trichomes and sub-nodal swellings. The leaves may be ovate, elliptical, lanceolate, or linear-lanceolate, arranged in a decussate opposite pattern. The flowers are aggregated in verticillasters positioned on the superior portions of the stems and branches [1,2,4,6].

Species of the genus *Galeopsis* are known by various Romanian vernacular names, including “zabră” (applied to *G. pubescens* and *G. speciosa*), “lungurică” (*G. tetrahit*), “tapoşnic” (*G. ladanum*), “cânepiţă” (*G. tetrahit*), and “faţa mâţei” (*G. angustifolia*). Some of these taxa are considered to be highly melliferous (notably *G. ladanum* and *G. tetrahit*), while others, such as *G. ladanum*, are also recognized as noxious weeds in cereal crop cultivation [2,4,6].

Depending on the species, plants of the genus *Galeopsis* are widely distributed across extensive areas in Romania, with some taxa being particularly common (*G. ladanum*, *G. speciosa*, and *G. tetrahit*). These species are found in ruderal habitats, forest edges, and shrubby areas (*G. bifida*); in forests, forest clearings, along tracks, and within hedgerows (*G. speciosa*); and along roadsides, in gardens, orchards, shrublands, forest clearings, or other ruderal sites (*G. tetrahit*) [2,4,6].

In the chemical composition of *Galeopsis* spp., flavonoids [3,7,8,9,10,11,12,13,14], phenolic acids [3,7,8], phenylpropanoid glycosides [3,7,8,15], iridoids [3,7,16,17,18], diterpenoids [3,7,19,20,21], triterpenoid compounds [3,7], essential oils [3,7,22,23], and fatty acids [3,7,24,25,26,27,28] have been identified.

Although few studies have examined the pharmacological activities of these plants, the genus *Galeopsis* is noted for its sedative, anticholinesterase, neuroprotective [3,7,29,30], antioxidant [3,7,31,32,33], anti-inflammatory [3,7], expectorant [3,7], astringent [3,7], diuretic [3,7], anti-anemic [3,7], and remineralizing [3,7] properties.

Some of these plants are employed in traditional medicine in certain regions. *G. ladanum* is reported to be used in Italy for the treatment of respiratory disorders through an infusion prepared from its leaves or flowers [7]. *G. bifida* is cited in Asian phytotherapy among various ethnic groups. In Tibet, the aerial parts of *G. bifida* are utilized in the form of a decoction to treat oral afflictions (stomatitis) and gastrointestinal disorders, including gastritis, ulcers, gastroenteritis, and inflammations affecting the esophagus, stomach, or intestines, as well as conjunctivitis, cystitis, and inflammatory conditions of the genital organs [3]. In the Far East, a tincture derived from the aerial parts of *G. bifida* is employed to stimulate the appetite, manage gastric ailments, and address epilepsy, while nomadic populations in northern Asia use the plant for the treatment of hepatic diseases [3].

There is evidence suggesting the potential for intoxication—manifesting as transient limb paralysis—following the consumption of fruits (with seed oil even being implicated) from *Galeopsis* spp. (*G. bifida*, *G. ladanum*, *G. speciosa*, and *G. tetrahit*) [3,34].

Our paper aimed to investigate for the first time the total phenolic content (TPC), total flavonoid content (TFC), and phenolic acid profile in the roots, aerial parts, and leaves from three wild-grown *Galeopsis* spp. (*G. bifida*, *G. speciosa*, and *G. tetrahit*) collected from the southwestern region of Romania, along with their in vitro antioxidant and neuroprotective potentials. The antioxidant activity of *Galeopsis* spp. was assessed using 2,2-diphenyl-1-picrylhydrazyl (DPPH) radical scavenging (half-maximal inhibitory concentration—IC_50_), 2,2′-azino-*bis*(3-ethylbenzothiazoline-6-sulfonic acid) (ABTS) radical scavenging (IC_50_), and ferric-reducing antioxidant power (FRAP; mM Fe^2+^ equivalents) assays. Acetylcholinesterase (AChE) inhibitory activity (IC_50_) was assessed using a microplate-based technique. Correlation analyses (Spearman’s and Pearson’s tests) were performed between TPC, TFC, and antioxidant and neuroprotective potentials. Also, the research provides new data for a better understanding of *Galeopsis* spp. from a therapeutic perspective.

## 2. Results

### 2.1. Total Polyphenols and Flavonoids

**Figure 1:**
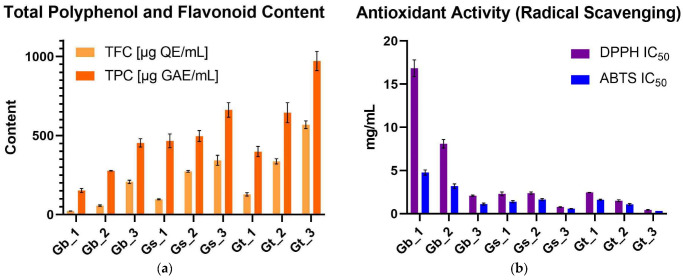

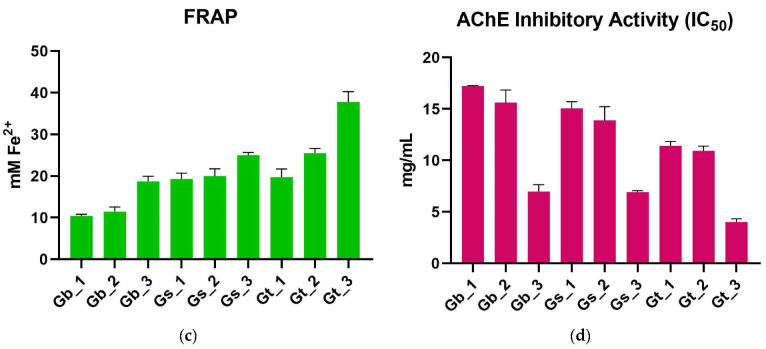
Bioactivity and phytochemical properties of *Galeopsis* samples: (**a**) TFC and TPC; (**b**) Antioxidant activity (radical scavenging); (**c**) FRAP assay; (**d**) AChE inhibitory assay. 1: Roots; 2: Aerial parts; 3: Leaves; ABTS: 2,2′-Azino-*bis*(3-ethylbenzothiazoline-6-sulfonic acid); AChE: Acetylcholinesterase; DPPH: 2,2-Diphenyl-1-picrylhydrazyl; FRAP: Ferric-reducing antioxidant power; GAE: Gallic acid equivalents; Gb: *G. bifida*; Gs: *G. speciosa*; Gt: *G. tetrahit*; IC_50_: Half-maximal inhibitory concentration; QE: Quercetin equivalents; TFC: Total flavonoid content; TPC: Total phenolic content.

**Figure 2:**
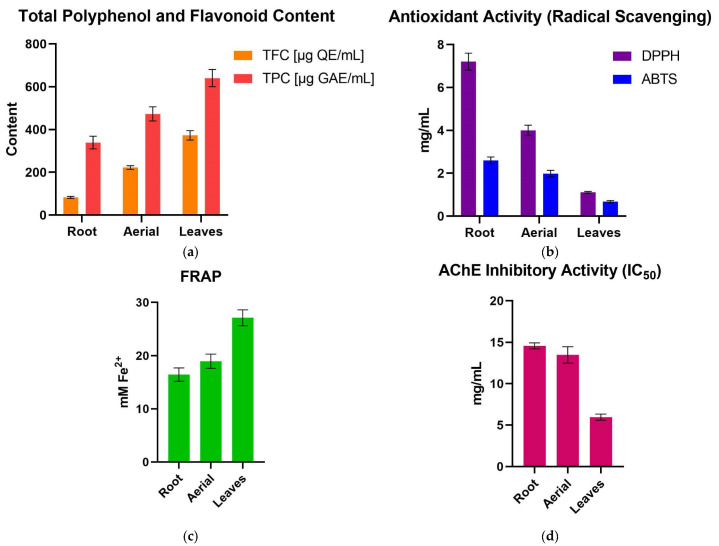
Bioactivity and phytochemical properties grouped by plant (*Galeopsis* spp.) part: (**a**) TFC and TPC; (**b**) Antioxidant activity (radical scavenging); (**c**) FRAP assay; (**d**) AChE inhibitory assay.

TPC and TFC were evaluated across the three *Galeopsis* spp. (*G. bifida*, *G. speciosa*, and *G. tetrahit*) and plant parts (roots, aerial parts, and leaves) (Table S1; Figure 1a and Figure 2a). The results from the two-way analysis of variance (ANOVA) demonstrated that both species and plant part significantly influenced TPC and TFC levels, with plant part showing a particularly strong effect on TFC.

#### 2.1.1. Effect of Species and Plant Part on TPC

The two-way ANOVA for TPC confirmed that species identity accounted for 47.57% (*p* < 0.0001) of the total variation, while plant part explained 41.98% (*p* < 0.0001). A significant interaction effect (8.31%) between species and plant part (*p* < 0.0001) indicated that the differences in TPC levels between the roots, aerial parts, and leaves were not consistent across species.

Post hoc analysis using Tukey’s test provided further insights into the differences among plant parts. Leaves exhibited the highest TPC level, significantly exceeding both aerial parts and roots (*p* < 0.0001), while aerial parts contained significantly more polyphenols than roots (*p* < 0.0001). The greatest contrast was observed between roots and leaves (mean difference: −356.2 μg gallic acid equivalents (GAE)/mL), highlighting the substantial polyphenol accumulation in leaf tissues (Figure 1a and Figure 2a).

Among species, *G. tetrahit* consistently displayed the highest TPC level across all plant parts, reaching 971.203 ± 60.377 μg GAE/mL in leaves, while *G. bifida* exhibited the lowest values, particularly in roots (152.674 ± 12.412 μg GAE/mL) (Figure 1a and Figure 2a).

These results confirmed that both species and plant part significantly contribute to polyphenol accumulation, with leaves being the richest source of polyphenols across all three species. The significant interaction effect suggested that the influence of plant part on TPC varies depending on species, emphasizing the importance of selecting the appropriate plant material for bioactive compound extraction.

#### 2.1.2. Effect of Species and Plant Part on TFC

A similar pattern was observed for TFC. The two-way ANOVA revealed that plant part had the strongest influence on TFC levels, explaining 51.99% of the total variation (*p* < 0.0001), while species accounted for 38.43% (*p* < 0.0001). A significant interaction effect (8.99%) was also detected, confirming that the impact of plant part on flavonoid content was species dependent.

Tukey’s post hoc test further highlighted the significant differences between plant parts. Leaves contained the highest TFC levels, significantly higher than both aerial parts and roots (*p* < 0.0001). Aerial parts had significantly more flavonoids than roots (*p* < 0.0001). The largest difference was between roots and leaves (mean difference: −290.3 μg quercetin equivalents (QE)/mL), further confirming that flavonoid accumulation was predominantly in leaves (Figure 1a and Figure 2a).

Among species, *G. tetrahit* had the highest flavonoid content across all plant parts, particularly in leaves (568.543 ± 24.174 μg QE/mL), while *G. bifida* exhibited the lowest values, particularly in roots (22.78 ± 0.663 μg QE/mL) (Figure 1a and Figure 2a).

The results clearly demonstrated that both species and plant part significantly influence the polyphenol and flavonoid contents, with leaves consistently exhibiting the highest levels across all three species. The significant interaction effect suggested that species-specific differences influence how polyphenols and flavonoids are distributed within different plant parts. These findings highlight the importance of selecting the appropriate species and plant material for maximizing the extraction of bioactive compounds with potential antioxidant and neuroprotective applications.

### 2.2. Antioxidant Activity (DPPH, ABTS, and FRAP)

DPPH (IC_50_), ABTS (IC_50_), and FRAP (mM Fe^2+^ equivalents) assays were used for the evaluation of *Galeopsis* spp. antioxidant activity (Table S1; Figure 1b,c and Figure 2b,c). The effects of species and plant part were analyzed using two-way ANOVA, which confirmed significant differences across both factors, along with an interaction effect.

#### 2.2.1. DPPH Radical Scavenging Activity

The DPPH radical scavenging assay revealed substantial differences in activity based on both species and plant part. Species had the strongest influence, accounting for 48.64% of the total variation (*p* < 0.0001), while plant part contributed 25.02% (*p* < 0.0001), with an interaction effect of 25.97% (*p* < 0.0001), confirming that the antioxidant potential varied not only between species but also among different plant parts within each species.

Among all samples, *G. bifida* roots exhibited the weakest DPPH radical scavenging activity, with an IC_50_ value of 16.84 ± 0.97 mg/mL, indicating a lower antioxidant potential. Conversely, *G. tetrahit* leaves had the highest radical scavenging activity, with an IC_50_ value of 0.458 ± 0.03 mg/mL, demonstrating a markedly stronger antioxidant effect. Leaves consistently showed the strongest DPPH activity across all species, with *G. speciosa* leaves also displaying a low IC_50_ value of 0.789 ± 0.03 mg/mL, comparable to *G. tetrahit*. Aerial parts exhibited an intermediate effect, with *G. bifida* aerial parts showing an IC_50_ of 8.102 ± 0.49 mg/mL, while *G. tetrahit* aerial parts demonstrated a stronger activity at 1.511 ± 0.11 mg/mL (Figure 1b and Figure 2b).

Post hoc comparisons confirmed that leaves had significantly higher radical scavenging activity than aerial parts and roots (*p* < 0.0001), and aerial parts showed stronger activity than roots (*p* < 0.0001).

#### 2.2.2. ABTS Radical Scavenging Activity

A similar trend was observed for the ABTS assay, where species explained 47.01% of the total variation (*p* < 0.0001), plant part accounted for 36.86% (*p* < 0.0001), and an interaction effect of 15.27% was found (*p* < 0.0001). These results confirmed that both genetic and morphological factors influenced the ABTS radical scavenging potential of *Galeopsis* spp.

The lowest ABTS radical scavenging activity was observed in *G. bifida* roots, which had an IC_50_ value of 4.772 ± 0.30 mg/mL, while the strongest activity was found in *G. tetrahit* leaves, with an IC_50_ of 0.328 ± 0.003 mg/mL. Among aerial parts, *G. speciosa* exhibited a slightly stronger effect (1.665 ± 0.10 mg/mL) compared to *G. bifida* (3.2 ± 0.25 mg/mL) (Figure 1b and Figure 2b).

Post hoc analysis showed that leaves exhibited significantly stronger ABTS radical scavenging activity than aerial parts and roots (*p* < 0.0001), with aerial parts also showing significantly higher activity than roots (*p* < 0.0001).

#### 2.2.3. FRAP Assay

Unlike DPPH and ABTS, where lower IC_50_ values indicate stronger radical scavenging, FRAP measures the reducing power, meaning higher values correspond to stronger antioxidant activity. The two-way ANOVA results demonstrated that species accounted for 55.42% of the total variation (*p* < 0.0001), plant part explained 34.28% (*p* < 0.0001), and the interaction effect contributed 7.861% (*p* < 0.0001), making species the dominant determinant of reducing power.

The highest FRAP value was recorded in *G. tetrahit* leaves, which exhibited 37.763 ± 2.52 mM Fe^2+^ equivalents, confirming the strongest reducing capacity. Conversely, *G. bifida* roots had the lowest FRAP activity at 10.392 ± 0.40 mM Fe^2+^ equivalents, aligning with the trend observed in the other assays. Aerial parts displayed moderate activity, with *G. speciosa* aerial parts showing a FRAP value of 19.979 ± 1.75 mM Fe^2+^ equivalents, while *G. tetrahit* aerial parts had a notably higher value at 25.480 ± 1.16 mM Fe^2+^ equivalents (Figure 1c and Figure 2c).

Post hoc comparisons revealed that leaves exhibited significantly higher reducing power than aerial parts and roots (*p* < 0.0001), and aerial parts also had significantly greater reducing power than roots (*p* = 0.0059).

These results confirmed that leaves consistently exhibited the strongest antioxidant potential across all three assays, while roots displayed the weakest activity in all cases. Among species, *G. tetrahit* consistently showed the highest antioxidant potential, followed by *G. speciosa*, while *G. bifida* exhibited the weakest activity.

### 2.3. Neuroprotective (AChE Inhibition) Activity

The AChE inhibition activity was assessed across the three *Galeopsis* spp. and the three plant parts to evaluate their potential neuroprotective effects. The results are expressed as IC_50_ values (mg/mL), where lower values indicate stronger AChE inhibition activity (Table S1; Figure 1d and Figure 2d). The two-way ANOVA confirmed that both species and plant part significantly influenced AChE inhibition, with plant part being the dominant factor.

The two-way ANOVA results revealed that plant part was the strongest determinant of AChE inhibition, accounting for 77.93% of the total variation (*p* < 0.0001). Species also played a significant role, explaining 18.88% of the variation (*p* < 0.0001). A minor but statistically significant interaction effect (1.368%, *p* = 0.0311) indicated that the effect of plant part on AChE inhibition was slightly dependent on species.

Among plant parts, leaves exhibited the strongest AChE inhibition activity, as indicated by the lowest IC_50_ values across all species. *G. tetrahit* leaves had the strongest inhibition, with an IC_50_ of 4.002 ± 0.32 mg/mL, followed by *G. speciosa* leaves (6.92 ± 0.14 mg/mL) and *G. bifida* leaves (6.97 ± 0.68 mg/mL). These lower IC_50_ values indicated high inhibitory activity in the leaf extracts (Figure 1d and Figure 2d).

By contrast, roots showed the weakest AChE inhibition activity, requiring higher concentrations to achieve 50% inhibition. *G. bifida* roots exhibited the highest IC_50_ value at 17.23 ± 0.04 mg/mL, followed by *G. speciosa* roots (15.06 ± 0.64 mg/mL) and *G. tetrahit* roots (11.42 ± 0.42 mg/mL) (Figure 1d and Figure 2d).

Aerial parts demonstrated moderate AChE inhibition, with *G. bifida* aerial parts having an IC_50_ of 15.63 ± 1.21 mg/mL, slightly higher than *G. speciosa* aerial parts (13.89 ± 1.33 mg/mL) and *G. tetrahit* aerial parts (10.94 ± 0.45 mg/mL) (Figure 1d and Figure 2d).

The post hoc Tukey’s multiple comparisons test revealed significant differences between plant parts. Leaves exhibited significantly stronger AChE inhibition (lower IC_50_) than aerial parts (*p* < 0.0001), with a mean difference of 7.523 mg/mL. Leaves also had significantly stronger AChE inhibition than roots (*p* < 0.0001), with a mean difference of 8.606 mg/mL, while aerial parts showed significantly stronger inhibition (lower IC_50_) than roots (*p* = 0.0128), confirming that root extracts were the least potent inhibitors.

These findings confirmed that AChE inhibition activity increased progressively from the roots to aerial parts to leaves, with leaves consistently exhibiting the strongest inhibitory effects across all species.

### 2.4. HPTLC Fingerprinting for Antioxidant and Neuroprotective Activities

**Figure 3:**
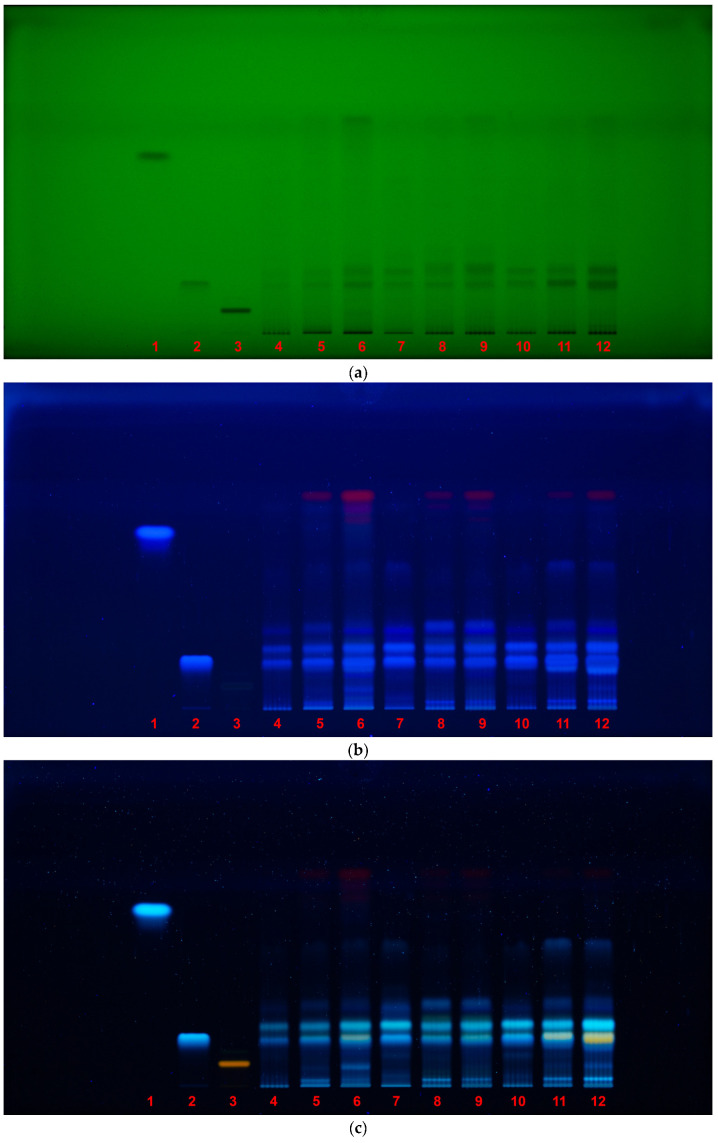

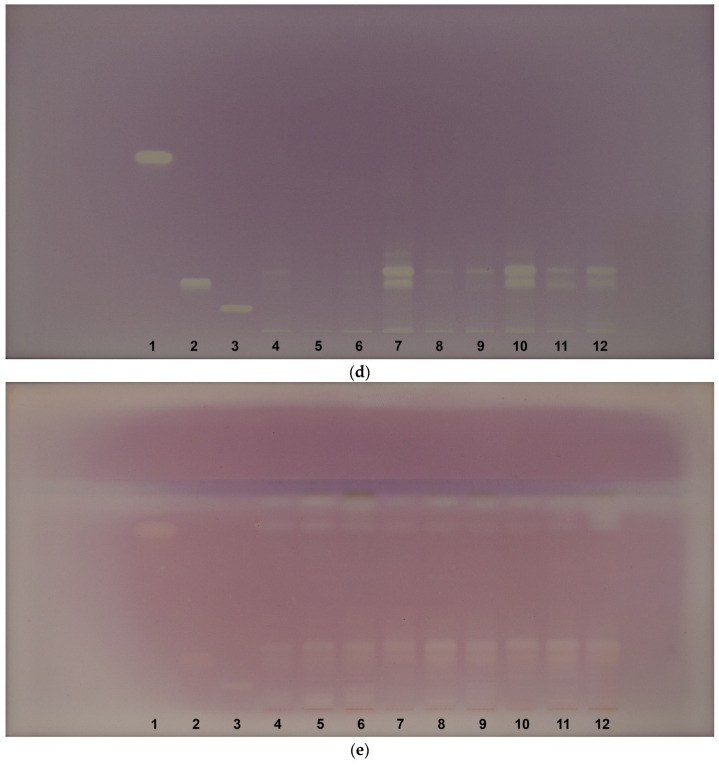
HPTLC fingerprint of *Galeopsis* spp. samples and reference standards: (**a**) 254 nm UV light, without derivatization; (**b**) 366 nm UV light, without derivatization; (**c**) 366 nm UV light, derivatization with NP–PEG reagent; (**d**) Antioxidant activity (DPPH assay, white light); (**e**) Neuroprotective activity (AChE inhibition assay, white light). Lanes: 1—Caffeic acid; 2–Chlorogenic acid; 3—Rutin; 4—*G. bifida* roots; 5—*G. bifida* aerial parts; 6—*G. bifida* leaves; 7—*G. speciosa* roots; 8—*G. speciosa* aerial parts; 9—*G. speciosa* leaves; 10—*G. tetrahit* roots; 11—*G. tetrahit* aerial parts; 12—*G. tetrahit* leaves.

High-performance thin-layer chromatography (HPTLC) was employed to obtain comparative phytochemical fingerprints and to visualize the antioxidant and neuroprotective activities of *Galeopsis* spp. extracts. The method allowed rapid screening and qualitative assessment of key phenolic and flavonoid compounds, as well as their associated biological activities via in situ DPPH radical scavenging and AChE inhibition assays (Table 1; Figure 3a–e).

Under 254 nm and 366 nm UV light, prominent bands were observed corresponding to phenolic acids and flavonoids. Chlorogenic acid (R_f_ 0.22) was clearly detected in all samples, appearing as dark bands under 254 nm and fluorescent spots at 366 nm, corroborating its presence and abundance as also confirmed by UHPLC analysis. Derivatization with NP–PEG reagent enhanced flavonoid fluorescence, particularly revealing a unique orange, fluorescent band in *G*. *tetrahit* leaves that was absent for other species or plant parts, suggesting the presence of a potentially unique flavonoid compound contributing to its high bioactivity.

Interestingly, although caffeic acid was included as a reference standard (R_f_ ≈ 0.79), no corresponding band was observed in the samples, despite being detected in low concentrations by UHPLC. This discrepancy may be attributed to the inherent limitations of HPTLC, which, while excellent for visualization and rapid screening, is less sensitive and specific than UHPLC. Its lower detection sensitivity may fail to reveal compounds present in trace amounts, while limited resolution can result in overlapping or co-eluting bands. The inhibition zone observed around R_f_ ≈ 0.79 in the AChE bioautography assay, despite the apparent absence of caffeic acid, suggested that another compound with similar chromatographic behavior may be responsible for the observed neuroactivity. This highlighted the need for complementary, high-resolution techniques such as liquid chromatography–tandem mass spectrometry (LC–MS/MS) or nuclear magnetic resonance (NMR) to isolate and identify such unknown bioactive constituents.

In the DPPH bioautography assay, distinct yellow bands were observed at R_f_ 0.22 in all samples, corresponding to chlorogenic acid and confirming its antioxidant role. Stronger DPPH and AChE inhibition zones were consistently observed in the leaf extracts, particularly from *G*. *tetrahit*, which aligned with the quantitative antioxidant and neuroprotective data presented earlier. This pattern further validated that leaf tissues, across all species, possessed the highest concentrations of biologically active compounds.

Overall, the HPTLC fingerprinting complemented the UHPLC quantification and spectrophotometric assays, confirming compound presence and activity trends, while also indicating the existence of yet unidentified compounds with potential pharmacological value. These findings reinforced the utility of HPTLC as a rapid, cost-effective screening tool and highlighted its value in guiding future isolation and structural elucidation efforts.

### 2.5. Phenolic Acids Profile (UHPLC Analysis)

The ultra-high-performance liquid chromatography (UHPLC) analysis of *Galeopsis* spp. revealed significant variations in phenolic acid composition across different plant parts. A total of eight phenolic acids were quantified based on their retention times, with gallic acid eluting first at 1.80 min, followed by protocatechuic acid (3.70 min), chlorogenic acid (5.83 min), vanillic acid (6.11 min), caffeic acid (6.36 min), syringic acid (6.64 min), *p*-coumaric acid (7.80 min), and ferulic acid (8.54 min) (Figure S1).

The results showed that chlorogenic acid was the most abundant compound, with the highest levels detected in *G. tetrahit* leaves (22,347.907 ± 1117.395 μg/g), followed by *G. tetrahit* aerial parts (11,678.509 ± 583.925 μg/g) and *G. speciosa* leaves (8712.628 ± 435.631 μg/g). By contrast, roots contained significantly lower levels of this compound, confirming that chlorogenic acid is concentrated in the aerial parts of these species (Table S2).

Among the other phenolic acids, *p*-coumaric acid and ferulic acid were particularly abundant in leaves, with *G. speciosa* leaves exhibiting the highest *p*-coumaric acid content (534.110 ± 26.706 μg/g), while *G. tetrahit* leaves had the highest ferulic acid concentration (271.089 ± 13.554 μg/g). Caffeic acid was detected in varying amounts, with *G. speciosa* leaves containing the highest levels (288.87 ± 14.444 μg/g). Notably, gallic acid was mostly absent in *G. bifida* and *G. speciosa* but was detected in *G. tetrahit* leaves (40.962 ± 2.048 μg/g). Similarly, protocatechuic acid was present in moderate concentrations, with the highest levels found in *G. tetrahit* leaves (176.536 ± 8.827 μg/g). The analysis also revealed that syringic acid and vanillic acid were detected across all species, with *G. speciosa* leaves containing the highest levels of vanillic acid (421.963 ± 21.098 μg/g) (Table S2).

Overall, leaves consistently exhibited the highest phenolic acid content, whereas roots contained the lowest concentrations. These findings supported the previous TPC and TFC results, highlighting leaves as the most bioactive plant part. The high concentration of chlorogenic acid, caffeic acid, and *p*-coumaric acid in leaves suggested that these compounds may contribute significantly to the strong antioxidant and neuroprotective activity observed in previous assays. Given the therapeutic potential of these bioactive compounds, future studies should focus on isolating and further characterizing these phenolic acids for their pharmacological applications.

### 2.6. Correlation

The antioxidant activities of *Galeopsis* spp. were assessed using DPPH, ABTS, and FRAP assays and are expressed as IC_50_ values (μg/mL) for DPPH and ABTS and as mM Fe^2+^ equivalents for FRAP. The normality of the data was evaluated using the Shapiro–Wilk test, which confirmed that ABTS and FRAP values followed a normal distribution, while DPPH values did not. Consequently, Spearman’s correlation analysis was applied to DPPH-related comparisons, while Pearson’s correlation analysis was used for ABTS vs. FRAP. The correlation analysis yielded the following results:

- DPPH IC_50_ vs. ABTS IC_50_: A strong positive correlation (*r* = 0.983, *p* < 0.05) was observed, indicating that extracts with higher radical scavenging efficiency in the DPPH assay also exhibited strong activity in the ABTS assay;
- DPPH IC_50_ vs. FRAP (mM Fe^2+^): A negative correlation (*r* = −0.833, *p* < 0.05) was found, suggesting that extracts requiring higher concentrations to inhibit 50% of DPPH radicals tended to exhibit higher reducing power in the FRAP assay;
- ABTS IC_50_ vs. FRAP (mM Fe^2+^): A negative correlation (*r* = −0.817, *p* < 0.05) was also observed, indicating an inverse relationship between radical scavenging capacity and ferric-reducing ability.

The strong correlation between DPPH and ABTS IC_50_ values suggested that both assays measured similar radical scavenging mechanisms, likely driven by polyphenolic compounds. Since lower IC_50_ values indicate higher antioxidant activity, the negative correlations suggested that extracts requiring lower concentrations for DPPH and ABTS inhibition also tended to exhibit stronger reducing power in the FRAP assay.

These findings highlighted the complexity of antioxidant mechanisms and reinforced the necessity of using multiple assays to obtain a comprehensive understanding of antioxidant potential.

To further examine the relationship between polyphenolic content and antioxidant activity, the correlation between TPC and the three antioxidant assays (DPPH IC_50_, ABTS IC_50_, and FRAP in mM Fe^2+^ equivalents) was assessed. The results revealed the following correlations:

- TPC vs. DPPH IC_50_: A strong negative correlation (*r* = −0.9333, *p* = 0.0007) was found, indicating that extracts with higher polyphenol content required lower concentrations to inhibit 50% of DPPH radicals, thus demonstrating stronger radical scavenging activity;
- TPC vs. ABTS IC_50_: A moderate negative correlation (*r* = −0.8833, *p* = 0.0031) was found, suggesting that higher polyphenol levels were associated with greater ABTS radical scavenging efficiency;
- TPC vs. FRAP (mM Fe^2+^ equivalents): A strong positive correlation (*r* = 0.9333, *p* = 0.0007) was found, indicating that extracts with higher polyphenol content exhibited greater ferric-reducing power.

To evaluate the contribution of flavonoids to antioxidant activity, the correlation between TFC and the three antioxidant assays (DPPH IC_50_, ABTS IC_50_, and FRAP in mM Fe^2+^ equivalents) was analyzed. The correlation analysis revealed the following relationships:

- TFC vs. DPPH IC_50_: A strong negative correlation (*r* = −0.9167, *p* = 0.0013) was found, indicating that extracts with higher flavonoid content required lower concentrations to inhibit 50% of DPPH radicals, confirming their potent radical scavenging capacity;
- TFC vs. ABTS IC_50_: A moderate negative correlation (*r* = −0.8833, *p* = 0.0031) was found, suggesting that an increase in flavonoid content was associated with improved ABTS radical scavenging efficiency;
- TFC vs. FRAP (mM Fe^2+^ equivalents): A strong positive correlation (*r* = 0.9333, *p* = 0.0007) was found, indicating that extracts with higher flavonoid content exhibited greater ferric-reducing power.

The neuroprotective potential of *Galeopsis* spp. was assessed through AChE inhibition activity, and its relationship with TPC and TFC was analyzed. The correlation analysis yielded the following results:

- AChE inhibition vs. TPC: A moderate negative correlation (*r* = −0.8266, *p* = 0.0060) was found, suggesting that extracts with higher total polyphenol content exhibited greater AChE inhibition. The 95% confidence interval (CI) ranged from −0.9624 to −0.3603, supporting the statistical robustness of this relationship;
- AChE inhibition vs. TFC: A moderate negative correlation (*r* = −0.8335, *p* = 0.0053) was found, indicating that an increase in flavonoid content was associated with stronger AChE inhibition. The 95% CI ranged from −0.9640 to −0.3793, reinforcing the reliability of the association.

Both correlations were statistically significant (*p* < 0.05) and suggested that polyphenols, particularly flavonoids, may play a role in the neuroprotective activity of these extracts. The negative correlation indicated that extracts with higher levels of polyphenols and flavonoids required lower concentrations to inhibit AChE, highlighting their potential as natural AChE inhibitors.

To further explore the relationship between AChE inhibition and antioxidant activity, the correlation between AChE inhibition and DPPH IC_50_, ABTS IC_50_, and FRAP (mM Fe^2+^ equivalents) was analyzed. The normality of the data was confirmed using the Shapiro–Wilk test, and Spearman’s correlation analysis was performed to assess statistical associations. The results of the correlation analysis revealed the following:

- AChE inhibition vs. DPPH IC_50_: A moderate positive correlation (*r* = 0.6887, *p* = 0.0402) was found, indicating that extracts with higher AChE inhibition also tended to require lower concentrations to scavenge 50% of DPPH radicals. However, the correlation was weaker compared to other parameters, as reflected by the 95% CI ranging from 0.04535 to 0.9283;
- AChE inhibition vs. ABTS IC_50_: A strong positive correlation (*r* = 0.8085, *p* = 0.0083) was found, suggesting that extracts with greater AChE inhibition demonstrated enhanced ABTS radical scavenging activity. The 95% CI (0.3117 to 0.9581) reinforced the statistical robustness of this relationship;
- AChE inhibition vs. FRAP (mM Fe^2+^ equivalents): A moderate negative correlation (*r* = −0.8238, *p* = 0.0063) was found, showing that extracts with higher AChE inhibition exhibited stronger reducing power. The negative correlation suggested that extracts with high AChE inhibition had greater ferric-reducing capacity, a trend supported by the 95% CI of −0.9617 to −0.3526.

These findings indicated a clear link between the neuroprotective and antioxidant activities in the analyzed *Galeopsis* spp. extracts. The positive correlations with DPPH and ABTS IC_50_ values suggested that extracts with stronger radical scavenging properties may also have neuroprotective potential. Meanwhile, the negative correlation with FRAP highlighted that reducing power may play an independent or complementary role in AChE inhibition.

## 3. Discussion

The findings of this study align closely with previous research on *Galeopsis* spp., particularly regarding their phytochemical composition, antioxidant potential, and neuroprotective activity [3,29,30,31].

The high concentrations of chlorogenic acid, *p*-coumaric acid, and ferulic acid observed in this study confirmed the polyphenol-rich nature of these plants, supporting previous reports that identified *G. bifida* as a source of phenylethanoid glycosides and flavone derivatives with strong antioxidant properties.

The findings also indicate that flavonoids are strongly localized in the leaves, aligning with their known role in plant defense and UV protection. The significant differences between species further emphasize the genetic variation in flavonoid biosynthesis among *Galeopsis* spp.

### 3.1. Comparison with Previous Literature

This study provides one of the most comprehensive investigations into the phytochemical composition and biological potential of *Galeopsis* spp. to date. While earlier studies have acknowledged the presence of flavonoids and other secondary metabolites in this genus [3,7,8,9,10,11,12,13,14], few have systematically compared different species or plant parts, and even fewer have quantified the phenolic acid profile alongside bioactivity assays.

Our results confirm that *Galeopsis* spp., particularly *G. tetrahit*, are rich in polyphenolic compounds. Chlorogenic acid was identified as the dominant phenolic acid, especially in leaf tissues, aligning with earlier research suggesting that chlorogenic acid plays a key role in the antioxidant and neuroprotective activities of members of the family *Lamiaceae* [31,32,33]. However, previous studies on *Galeopsis* spp. have largely focused on flavone aglycones, iridoids, and phenylethanoid glycosides, without detailed phenolic acid quantification. This study fills that gap, providing new data that more precisely links individual phenolic acids with specific bioactivities.

The strong antioxidant activity demonstrated in the DPPH, ABTS, and FRAP assays is consistent with earlier reports on related genera such as *Lamium*, *Salvia*, and *Melissa*, which also exhibit high phenolic content and radical scavenging capacity [3,7,31]. Our findings expand this knowledge to *Galeopsis* spp. and show that these properties are highly dependent on the plant part analyzed. The leaves consistently exhibited the highest levels of total phenolics, total flavonoids, and antioxidant activity, which aligns with the known localization of these compounds in aerial tissues for UV protection and oxidative stress defense.

In terms of neuroprotective potential, our results confirm that *Galeopsis* spp. extracts—particularly from the leaves—have significant AChE inhibitory activity. This aligns with traditional uses of *Galeopsis* spp. for sedative or cognitive-supportive effects and with recent findings that hydroxycinnamic acids, flavonoids, and iridoid glycosides present in the genus can influence cholinergic signaling [29,30]. The consistent increase in AChE inhibition from the roots to aerial parts to leaves across all species further suggests a physiological gradient in bioactive compound distribution.

Although *Galeopsis* spp. have been acknowledged in an ethnomedicinal context, prior scientific literature remains fragmented, with most studies limited to partial screenings of specific secondary metabolite classes or single-species analyses. Phenolic acid profiling and functional correlation with biological activity have been largely neglected. To our knowledge, this is the first study to quantitatively assess multiple phenolic acids across three *Galeopsis* spp. and distinct plant parts, while directly linking them to antioxidant and neuroprotective activities.

Our multi-method approach—including spectrophotometric assays, UHPLC quantification, and HPTLC fingerprinting—allows for a more nuanced characterization of each species’ phytochemical profile. Importantly, this study establishes clear associations between specific phenolic acids (e.g., chlorogenic, *p*-coumaric, and ferulic acids) and both antioxidant and AChE inhibitory activities.

Overall, this study significantly broadens our understanding of the phytochemical and pharmacological landscape of *Galeopsis* spp. and positions them as promising candidates for the development of plant-based antioxidant and cognitive-supportive formulations.

### 3.2. Total Polyphenols and Flavonoids

The relationship between TPC and TFC was evaluated using Pearson’s correlation analysis, following confirmation of normal data distribution via the Shapiro–Wilk test. The results indicated a strong positive correlation between TPC and TFC (*r* = 0.9653, *p* < 0.0001), demonstrating a significant association between these two parameters.

This finding suggests that flavonoids constitute a major portion of the total polyphenolic content in the analyzed *Galeopsis* spp. The high correlation implies that an increase in TFC is accompanied by a proportional increase in TFC, reinforcing the contribution of flavonoids to the overall phytochemical profile. It is important to note that the aluminum chloride (AlCl_3_) colorimetric assay used for TFC determination is prone to false-positive results, primarily because it can react not only with flavonoids but also with non-flavonoid phenolic compounds, such as certain phenolic acids, tannins, and other interfering substances. These non-flavonoids are capable of forming similar complexes with AlCl_3_, which can artificially increase the estimated flavonoid content [35].

Given the well-documented biological activities of flavonoids, these results highlight their potential role in the antioxidant and neuroprotective effects of the extracts.

### 3.3. Antioxidant Activity

The antioxidant activity results are consistent with earlier studies that demonstrated that *Galeopsis* spp. possess strong DPPH and ABTS radical scavenging potentials, along with the FRAP assay [3,30,31,32,33]. These effects have been previously linked to phenylethanoid glycosides and flavonoid glycosides, particularly luteolin and apigenin derivatives. The current study further supports these findings by establishing strong correlations between TPC, TFC, and antioxidant activity, indicating that these compounds are the primary contributors to the radical scavenging potential of *Galeopsis* spp. extracts. The HPTLC fingerprinting of DPPH activity further corroborated these results, showing that chlorogenic acid, along with flavonoid-related compounds, was responsible for the observed antioxidant effects.

These findings confirm that polyphenols play a key role in the antioxidant activity of *Galeopsis* spp., contributing both to radical scavenging (DPPH, ABTS) and reducing power (FRAP). The observed negative correlations with DPPH and ABTS IC_50_ values indicated that extracts with higher TPC exhibited lower IC_50_ values, confirming their enhanced ability to neutralize free radicals. Conversely, the strong positive correlation between TPC and FRAP implied that polyphenols were also effective electron donors, reinforcing their reducing capacity.

The results suggest that flavonoids significantly contribute to both radical scavenging activity and reducing power in the tested *Galeopsis* spp. The negative correlations with DPPH and ABTS IC_50_ values indicated that flavonoid-rich extracts exhibited stronger antioxidant activity, requiring lower concentrations to achieve 50% inhibition. The strong positive correlation between TFC and FRAP further supported the role of flavonoids as efficient electron donors, reinforcing their involvement in redox reactions.

### 3.4. Neuroprotective Activity

Regarding neuroprotective activity, the results of this study reinforce previous findings on the AChE inhibitory potential of *Galeopsis* spp. Earlier research identified several bioactive metabolites with AChE inhibitory properties, including iridoid glycosides (harpagide, harpagide 8-O-acetate, ajugoside), phenylethanoid glycosides (verbascoside, isoverbascoside), flavonoid glycosides (luteolin and apigenin derivatives), and hydroxycinnamic acids (caffeoylquinic acids, e.g., chlorogenic acid) [3,7,8,29,30]. The current study confirmed that chlorogenic acid was present in all samples and exhibited moderate AChE inhibition, supporting its role as a neuroprotective agent. Additionally, an unknown compound at the same R_f_ as caffeic acid exhibited inhibitory activity in the AChE HPTLC assay, suggesting the presence of another bioactive metabolite contributing to neuroprotection. The strongest AChE inhibition zones were observed in *G. tetrahit* leaves, which also exhibited a unique orange-fluorescent flavonoid that was not present in the other species. The results suggest that *G. tetrahit* may contain distinct neuroactive flavonoids that warrant further investigation.

These findings align with previous research suggesting that polyphenolic compounds, including flavonoids, can modulate cholinergic activity and contribute to neuroprotective effects. Further investigations into specific bioactive compounds responsible for AChE inhibition could provide deeper insights into their potential application in managing neurodegenerative conditions.

The strong association between AChE inhibition and antioxidant activity aligns with existing research suggesting that oxidative stress is closely linked to neurodegenerative diseases and that antioxidants may exert neuroprotective effects by reducing oxidative damage and modulating cholinergic activity. Future studies focusing on specific bioactive compounds with dual antioxidant and neuroprotective activities could provide further insights into their mechanisms of action and potential therapeutic applications.

Overall, the findings of this study confirm and expand upon previous research, reinforcing the high polyphenol and flavonoid contents of *Galeopsis* spp., particularly in leaves. The strong antioxidant and neuroprotective activity observed in *G. tetrahit* and *G. speciosa* leaves suggests that these plants contain valuable bioactive compounds with potential therapeutic applications in oxidative stress-related and neurodegenerative diseases. Future research should focus on isolating and characterizing the specific compounds responsible for these effects to better understand their pharmacological potential.

### 3.5. Study Limitations

The study’s findings on *Galeopsis* spp. should be interpreted in light of several methodological limitations. The use of ultrasound-assisted extraction with 70% ethanol, while efficient for extracting polyphenols and flavonoids, may have resulted in the underrepresentation of other potentially bioactive compound classes, such as alkaloids, lipophilic terpenes, or polysaccharides, due to specific solvent selectivity; future work could benefit from employing multiple extraction techniques to capture a broader chemical profile. Furthermore, compound identification through HPTLC fingerprinting and UHPLC quantification relied on comparison with reference standards, meaning that unknown compounds—like one observed at the same R_f_ value as caffeic acid in AChE inhibition assays—were not structurally elucidated, necessitating the use of more advanced analytical methods like LC–MS/MS or NMR spectroscopy for definitive identification and the discovery of novel molecules. Additionally, the assessment of biological activity was confined to in vitro assays (including DPPH, ABTS, and FRAP antioxidant tests and AChE inhibition assays), which, while indicative of potential, do not always translate to in vivo efficacy due to complex factors like bioavailability, metabolism, and cellular interactions, underscoring the need for validation through cell-based models, animal studies, and pharmacokinetic analyses. Finally, the AlCl_3_ colorimetric method used for quantifying TFC is susceptible to interference, as certain phenolic acids and other non-flavonoid compounds can react with the reagent, potentially leading to an overestimation of flavonoid levels; employing more specific quantification techniques, such as UHPLC–MS/MS, would provide more accurate validation.

## 4. Materials and Methods

### 4.1. Plant Material

The roots, aerial parts, and leaves of wild-grown *Galeopsis* spp. were harvested during the flowering stage (July–August 2024) from distinct ecological zones in southwestern Romania. *G*. *bifida* and *G*. *speciosa* were harvested near Tismana City, Gorj County, at GPS coordinates 45°05′23.8″ N, 22°55′06.2″ E and 45°05′18.2″ N, 22°55′06.1″ E, respectively. *G*. *tetrahit* was collected from Lăpuşnicel Village, Caraş-Severin County, at 44°59′17.4″ N, 22°13′50.9″ E.

These regions are characterized by a temperate-continental climate with warm summers and moderate precipitation. Average July temperatures range between 20 and 23 °C, and the monthly rainfall averages 60–80 mm, creating favorable conditions for the biosynthesis of secondary metabolites. According to regional soil surveys, these areas contain predominantly luvisols and cambisols—soils known for moderate acidity, good drainage, and relatively high organic content, supporting herbaceous plant diversity and productivity [36,37].

Soil properties and climate are well-established factors influencing phytochemical expression in plants. Nutrient availability, pH, and water retention in the soil can stimulate the synthesis of polyphenols and flavonoids as stress response metabolites. This is particularly relevant for species in the family *Lamiaceae*, where abiotic stress such as increased UV exposure and moderate drought can trigger enhanced accumulation of antioxidant compounds [38,39]. Thus, the edaphoclimatic conditions of the collection sites likely contributed to the observed variation in phenolic acid concentration and bioactivity between species and plant parts.

Although the regions and timing of collection were selected with phytochemical richness in mind, we note that the plant material was not consistently harvested at peak flowering stage, particularly in the case of *G. tetrahit*. Due to the scattered and less predictable natural occurrence of this species, it required extended field effort to locate viable populations, which resulted in some individuals being past their full reproductive stage at the time of collection. Consequently, the goal of this study was not to evaluate seasonal or phenological variation but rather to establish a foundational phytochemical and bioactivity profile across three *Galeopsis* spp. and distinct plant tissues in their natural ecological context. The consistent patterns observed—such as higher polyphenol content and bioactivity in leaves across all species—suggest that species- and tissue-level variation is robust despite minor differences in phenological timing. Now that these specific ecological zones have been identified and characterized, future studies can build upon this work by incorporating month-to-month comparisons and phenological stage analysis to better understand how seasonal factors modulate secondary metabolite accumulation.

The plant material for analysis was stored in the Herbarium of the Department of Pharmaceutical Botany, Faculty of Pharmacy, University of Medicine and Pharmacy of Craiova. The plant material was air-dried and deposited in brown paper bags at room temperature (RT) in a cool and dark area 24 h before processing for extraction and analysis. Our research did not involve endangered or protected plant species.

A systematic notation representing different *Galeopsis* spp., their respective vegetal products, date/site of collection, and voucher specimens was used in this study. A clear and organized reference to the specific plant species and parts analyzed in the experiments was facilitated by this notation (Table 2).

### 4.2. Chemicals and Reagents

The solvents used in this study included ethanol, methanol, acetonitrile, and ethyl acetate (Merck, Darmstadt, Germany). Ultrapure water was obtained using a HALIOS 6 lab water system (Neptec, Montabaur, Germany) to ensure the required purity for aqueous solutions and dilutions. For UHPLC analysis, formic acid (Merck) was used as an additive to enhance the performance of the mobile phases.

The reagents selected to support the experimental assays included Folin–Ciocalteu reagent, sodium carbonate, DPPH, ABTS, potassium persulfate, sodium acetate, acetic acid, 2,4,6-*tris*(2-pyridyl)-1,3,5-triazine (TPTZ), quercetin, natural products–polyethylene glycol (NP–PEG) reagent, ferric chloride (FeCl_3_), ferrous sulfate heptahydrate (FeSO_4_·7H_2_O), and hydrochloric acid (HCl) (Sigma-Aldrich, Taufkirchen, Germany). These reagents were used for the determination of TPC, antioxidant activity, and enzymatic assays. For TPC, Folin–Ciocalteu reagent was used together with sodium bicarbonate. AlCl_3_ from Sigma-Aldrich was specifically used for the TFC assay.

For the AChE inhibition assay, the primary reagents included AChE from *Electrophorus electricus*, 1-naphthyl acetate, Fast Blue B salt, Tris-HCl buffer solution (pH 7.8, 0.05 M), and rivastigmine as a positive control (Sigma-Aldrich).

In UHPLC analysis, a set of phenolic acid standards—including caffeic acid, chlorogenic acid, *p*-coumaric acid, ferulic acid, gallic acid, protocatechuic acid, syringic acid, and vanillic acid (Merck Millipore, Darmstadt, Germany)—was used for calibration and compound identification.

For HPTLC analysis, Silica gel 60 F_254_ glass plates (20 × 10 cm) were obtained from Merck (Darmstadt, Germany).

### 4.3. Extraction Procedure

The extraction of plant material was carried out using an ultrasound-assisted extraction method, with 70% ethanol as the solvent. A measured quantity of 1 g of finely ground plant material was combined with 10 mL of the ethanol solution in an appropriate container. The mixture underwent ultrasonic treatment in a Bandelin Sonorex Digiplus DL 102H ultrasound bath (Bandelin electronic GmbH & Co. KG, Berlin, Germany) operating at 100 W power and a frequency of 35 kHz for 20 min at a controlled temperature of 50 °C. The application of ultrasonic waves facilitated the breakdown of plant cell walls, enhancing the release of bioactive compounds into the solvent.

Following extraction, the solution was filtered through a 0.22 μm syringe filter equipped with a water wettable polytetrafluoroethylene (WWPTFE) membrane (Acrodisc, Pall Corporation, Port Washington, NY, USA) to separate the liquid extract from any residual solid material. The obtained extract was subsequently used for TPC and TFC determination, as well as antioxidant and neuroprotection assays.

For UHPLC analysis, 1 mL of the extract was carefully evaporated under a gentle nitrogen stream to eliminate the solvent. The dried residue was then reconstituted in a mixture of water and acetonitrile (9:1, *v*/*v*) to ensure compatibility with the UHPLC mobile phase system. This step was essential for optimizing the dissolution of bioactive compounds prior to chromatographic separation and detection. Before injection into the UHPLC system, the reconstituted solution was filtered through a 0.22 μm syringe filter to remove any particulate matter [40].

### 4.4. Standards Preparation

Caffeic acid, chlorogenic acid, *p*-coumaric acid, ferulic acid, gallic acid, protocatechuic acid, syringic acid, and vanillic acid were used as standards for the UHPLC analysis. A stock solution of each standard was prepared at 1 mg/mL concentration using methanol. To achieve calibration concentrations ranging from 0.1 μg/mL to 50 μg/mL, serial dilutions were made. For both standards and samples, a volume of 10 μL was injected into the UHPLC system.

### 4.5. Total Polyphenols and Flavonoids

#### 4.5.1. TPC Assay

TPC was quantified using the Folin–Ciocalteu method, in a 96-well microplate format. Twenty microliters (20 μL) of the plant extract were pipetted into each well, followed by the addition of 100 μL of Folin–Ciocalteu reagent. The mixture was allowed to react for three minutes, after which 80 μL of 4% sodium carbonate solution was added. The microplate was stirred for another three minutes to ensure homogeneity. To facilitate color development, the reaction mixture was incubated in the dark for two hours. Following incubation, absorbance was measured at 620 nm using a FLUOstar Optima microplate reader (BMG Labtech, Ortenberg, Germany). A gallic acid standard curve (*y* = 0.003058*x* − 0.06987, *R*^2^ = 0.9912) was prepared with calibration solutions ranging from 5 mg/mL to 625 μg/mL to enable the quantification of the phenolic compounds in the extracts, expressed as μg GAE per mL of plant extract. Each measurement was performed in triplicate to ensure accuracy and reproducibility [41].

#### 4.5.2. TFC Assay

TFC was assessed using the AlCl_3_ colorimetric assay. A quercetin standard curve (*y* = 0.008505*x* + 0.05493, *R*^2^ = 0.9885) was prepared in 96% ethanol, with concentrations ranging from 30 to 100 μg/mL. For each assay, 50 μL of plant extract or quercetin standard solution was added to a 96-well microplate, followed by the addition of 10 μL of 10% AlCl_3_ solution. To this mixture, 150 μL of 96% ethanol was added, followed by 10 μL of 1 M sodium acetate. A blank control was prepared using 96% ethanol in place of the sample. After thorough mixing, the reaction was incubated for 40 min at RT in the dark. Absorbance was recorded at 410 nm using a FLUOstar Optima microplate reader (BMG Labtech, Ortenberg, Germany). The results are expressed as μg QE per mL of plant extract. Each sample was analyzed in triplicate to ensure reproducibility [41,42].

### 4.6. Antioxidant Activity Assays

#### 4.6.1. DPPH Antioxidant Assay

The DPPH radical scavenging assay was conducted by adding 50 μL of each sample to a 96-well microplate, followed by serial dilutions to obtain a gradient of decreasing concentrations. Next, 150 μL of a 0.2 mM DPPH solution in ethanol was added into each well. The reaction mixtures were incubated in the dark for 30 min at RT, after which the absorbance was measured at 517 nm using a FLUOstar Optima microplate reader (BMG Labtech). The antioxidant potential was evaluated by calculating the half-maximal inhibitory concentration (IC_50_), which represented the concentration required to scavenge 50% of the DPPH radicals. Each sample was analyzed in triplicate to ensure accuracy [41].

#### 4.6.2. ABTS Antioxidant Assay

In the ABTS radical scavenging assay, 50 μL of each sample was added to a 96-well microplate, followed by serial dilutions in the same manner as the DPPH assay. Then, 150 μL of ABTS reagent, prepared by mixing 7.4 mM ABTS with 2.6 mM potassium persulfate, was added to each well. After a reaction time of six minutes, the absorbance was measured at 620 nm using a FLUOstar Optima microplate reader (BMG Labtech). The IC_50_ value, representing the sample concentration necessary to inhibit 50% of the ABTS radicals, was determined from a dose–response curve. Each sample was tested in triplicate [40].

#### 4.6.3. FRAP Antioxidant Assay

The FRAP assay was performed by preparing fresh FRAP reagent consisting of acetate buffer (pH 3.6), 10 mM TPTZ solution in 40 mM HCl, and 20 mM FeCl_3_ solution. A calibration curve (*y* = 0.0006120*x* + 0.05580, *R*^2^ = 0.9976) was established using Fe^2+^ standards in the range of 65 to 500 μM. In each assay, 10 μL of the sample or standard was added to a 96-well microplate, followed by 190 μL of freshly prepared FRAP reagent. The reaction mixtures were incubated for 30 min at RT, after which the absorbance was recorded at 595 nm. The results are expressed as μmol Fe^2+^ equivalents, and all analyses were carried out in triplicate to ensure reliability [40].

### 4.7. Neuroprotective Activity Assay

The AChE inhibitory activity was assessed using a microplate-based assay, with each sample tested in triplicate to ensure reliability. The assay aimed to evaluate the ability of the test samples to inhibit AChE activity across a range of concentrations. Each sample underwent serial dilution directly on a 96-well microplate, starting from the stock extract solution, to generate a concentration gradient. To initiate the reaction, 50 μL of 1-naphthyl acetate solution (3 mg/mL in ethanol) was added to each well, serving as the enzymatic substrate. This was followed by the addition of 150 μL of AChE solution (3.33 U/mL) to catalyze the reaction, leading to the formation of measurable enzymatic products. To facilitate the detection of enzyme activity, 50 μL of Fast Blue B salt solution (3 mg/mL in water) was introduced into each well. This reagent reacts with the enzymatic products, producing a distinct color change that correlates with AChE activity. Rivastigmine (1 mg/mL in methanol), a known AChE inhibitor, was included as a positive control to establish a reference for the inhibitory potential of the test samples. Absorbance was recorded at 595 nm using a FLUOstar Optima microplate reader (BMG Labtech), and the collected data were analyzed to determine the IC_50_ value for each sample, indicating the concentration required to inhibit 50% of AChE activity [40].

### 4.8. HPTLC Fingerprinting for Antioxidant and Neuroprotective Activity

HPTLC fingerprinting was performed to assess the antioxidant potential (DPPH assay) and AChE inhibitory activity of the plant extracts [43]. Caffeic acid, chlorogenic acid, and rutin were used as reference standards.

Sample application was carried out using a Linomat 5 applicator, where 2 μL of each extract and standard was applied to the HPTLC plates. The chromatographic separation was conducted in a twin trough chamber using a mobile phase consisting of ethyl acetate, formic acid, and water (90:6:9, *v*/*v*/*v*). Prior to development, the chamber was saturated for 20 min to ensure optimal separation conditions. The plates were developed up to a solvent front position of 7 cm.

For the AChE inhibition assay, the plate was sprayed using the CAMAG Derivatizer (CAMAG, Muttenz, Switzerland) with 0.5 mL Tris-HCl buffer solution (pH 7.8, 0.05 M) used for prewetting and then 1.5 mL AChE solution (6.66 U/mL), after which the plate was sprayed with 0.5 mL of the 1:1 substrate/chromogenic reagent mixture (ethanolic 1-naphthyl acetate solution and aqueous Fast Blue B salt solution, 3 mg/mL each) and dried (three min).

Following development, the plates were air-dried at RT for 10 min before analysis. Visualization was conducted at 254 nm and 366 nm without derivatization, as well as post-derivatization using NP–PEG reagent at 366 nm and for DPPH and AChE in white light.

This method enabled the identification of bioactive compounds within the extracts based on their retention factor (R_f_) values and corresponding color changes indicative of antioxidant and neuroprotective properties [43].

### 4.9. UHPLC Analysis of Phenolic Acids

UHPLC analysis was performed using a Waters Acquity Arc system, equipped with a photodiode array (PDA) detector and a QDa mass detector (Waters, Milford, Massachusetts, USA). Chromatographic separation was achieved using a CORTECS C18 column (4.6 × 50 mm, 2.7 μm particle size), which was maintained at a temperature of 30 °C.

The mobile phase consisted of water with 0.01% formic acid (A) and acetonitrile with 0.01% formic acid (B). The gradient elution program was initiated with 99% A at a constant flow rate of 0.8 mL/min, which was held for one minute. Between 1 and 13 min, the proportion of mobile phase A was gradually reduced to 70%, which remained unchanged until 13.10 min. From 13.60 to 17.60 min, the composition shifted to 20% A, allowing for column cleaning and the removal of strongly retained compounds. The mobile phase then returned to its initial condition of 99% A at 18.10 min, and this was maintained until 21.10 min for system re-equilibration before the next injection. To ensure analytical stability and reproducibility, the column was equilibrated for 10 min between injections. Throughout the analysis, samples were kept at 8 °C to preserve their integrity.

For quantification, absorbance detection was set at 265 nm for gallic acid, protocatechuic acid, vanillic acid, and syringic acid, while 325 nm was used for chlorogenic acid, caffeic acid, *p*-coumaric acid, and ferulic acid. Mass confirmation was carried out in negative ion mode, targeting the following specific mass-to-charge (*m*/*z*) ratios: 153 (protocatechuic acid), 163 (*p*-coumaric acid), 167 (vanillic acid), 169 (gallic acid), 179 (caffeic acid), 193 (ferulic acid), 197 (syringic acid), and 353 (chlorogenic acid) [40,43].

### 4.10. Statistical Analysis

All experimental data were analyzed using GraphPad Prism 9 (GraphPad Software, version 9.0.2, San Diego, CA, USA). The results are expressed as mean ± standard deviation (SD), with all experiments performed in triplicate (*n* = 3). The normality of the data was assessed using the Shapiro–Wilk test, and based on the results, appropriate statistical tests were applied.

For comparisons between *Galeopsis* spp. (*G. bifida*, *G. speciosa*, and *G. tetrahit*) and plant parts (roots, aerial parts, and leaves), a two-way ANOVA was performed to determine the influence of these factors on TPC, TFC, antioxidant activity (DPPH, ABTS, and FRAP assays), and AChE inhibition. The post hoc Tukey’s multiple comparisons test was conducted to identify statistically significant differences between groups.

For correlation analyses, Pearson’s correlation coefficient (*r*) was used when the data followed a normal distribution, while the Spearman’s correlation test was applied for non-normally distributed data. Correlations were evaluated between TPC, TFC, and bioactivity assays (antioxidant and AChE inhibition tests) to determine potential relationships between the polyphenolic content and biological activity. The significance threshold was set at α = 0.05, with results considered statistically significant at *p* < 0.05, highly significant at *p* < 0.01, very highly significant at *p* < 0.001, and extremely significant at *p* < 0.0001.

All statistical tests were conducted in accordance with standard biostatistical methodologies, ensuring robust and reproducible data interpretation.

## 5. Conclusions

This study provides a comprehensive analysis of the phytochemical composition, antioxidant activity, and neuroprotective potential of three *Galeopsis* spp. (*G. bifida*, *G. speciosa*, and *G. tetrahit*). The results confirm that leaves contain the highest concentrations of phenolic acids and flavonoids, particularly chlorogenic acid, *p*-coumaric acid, and ferulic acid, which were identified as major bioactive compounds. Strong antioxidant activity was demonstrated through DPPH, ABTS, and FRAP assays, with leaves, particularly those of *G. tetrahit*, exhibiting the greatest radical scavenging potential. Additionally, AChE inhibition assay revealed that *G. tetrahit* leaves exhibited the strongest neuroprotective effects, which may be attributed to their high phenolic acid and flavonoid contents.

These findings align with previous research on *Galeopsis* spp., reinforcing their potential as natural sources of antioxidants and neuroprotective agents. However, this study represents one of the few in-depth investigations into the phytochemistry and bioactivity of these species, highlighting the need for further research on compound isolation, structural characterization, and in vivo validation. The presence of an unknown neuroactive compound at the same R_f_ as caffeic acid in the AChE inhibition assay suggests that *Galeopsis* spp. may contain previously unidentified bioactive molecules, warranting additional pharmacological exploration.

Overall, this study supports the medicinal relevance of *Galeopsis* spp., particularly in applications related to oxidative stress and neurodegenerative disorders. Future work should focus on elucidating the mechanisms of action, exploring their clinical relevance, and assessing the safety profile of these bioactive compounds to unlock their full therapeutic potential.

## Figures and Tables

**Table 1:** HPTLC fingerprint of *Galeopsis* spp. samples and reference standards.

HPTLC Fingerprint	Description
254 nm UV light, without derivatization	• Under shortwave UV light, dark bands indicate the presence of UV-absorbing compounds, such as phenolic acids and flavonoids; • Caffeic acid (R_f_ 0.79) was not detected in any of the samples, confirming its absence or presence at undetectable concentrations (low amount demonstrated by UHPLC assay); • Chlorogenic acid (R_f_ 0.22) was visible in all samples, confirming it as a major component of *Galeopsis* spp.; • Rutin (R_f_ 0.085) was detected as a dark band only as a reference.
366 nm UV light, without derivatization	• Under longwave UV light, compounds such as phenolic acids emit fluorescence, revealing their presence; • Chlorogenic acid (R_f_ 0.22) was again observed in all samples, confirming its stability and prevalence across species; • Rutin (R_f_ 0.085) was not visible under this condition, indicating that it does not fluoresce strongly without derivatization.
366 nm UV light, derivatization with NP–PEG reagent	• NP–PEG derivatization enhances flavonoid fluorescence (orange/yellow), allowing for clearer visualization; • Rutin (R_f_ 0.085) became visible after derivatization, confirming that its detection requires NP-PEG treatment; • Strong flavonoid fluorescence was observed in *G. tetrahit* leaves, with a unique, orange-colored band that was absent in other species and plant parts, but not at the same R_f_ as rutin.
Antioxidant activity (DPPH assay, white light)	• The DPPH assay was used to detect antioxidant activity, where active compounds appear as yellow bands against a purple background, indicating free radical scavenging activity; • Chlorogenic acid (R_f_ 0.22) correlated strongly with antioxidant activity, as yellow bands were observed at this R_f_ across all samples; • For the DPPH HPTLC assay, extracts from aerial parts and leaves were diluted five-fold to prevent oversaturation of the plate and ensure accurate visualization of antioxidant activity.
Neuroprotective activity (AChE inhibition assay, white light)	• The AChE inhibition assay was used to detect neuroprotective compounds, where active inhibitors appeared as clear bands against a purple background; • Chlorogenic acid (R_f_ 0.22) demonstrated visible AChE inhibition in all samples, suggesting that it may contribute to the neuroprotective effects observed in *Galeopsis* spp.; • A distinct inhibition zone appeared at R_f_ 0.79, the same Rf as caffeic acid; however, since caffeic acid was not detected in the chemical fingerprinting, this suggested the presence of another compound with neuroprotective properties that migrates similarly; • Slightly stronger inhibition zones were observed in *G. tetrahit* leaves, further reinforcing that this plant part contains potent neuroprotective compounds.

AChE: Acetylcholinesterase; DPPH: 2,2-Diphenyl-1-picrylhydrazyl; HPTLC: High-performance thin-layer chromatography; NP–PEG: Natural products–polyethylene glycol; UHPLC: Ultra-high-performance liquid chromatography; UV: Ultraviolet.

**Table 2:** Sample coding of plant material (*Galeopsis* spp.).

Sample	Species/Vegetal Product	Date/Collection Site (Southwest Romanian Flora; GPS Coordinates)	Voucher Specimen
Gb_1	*G. bifida*/*radix*	19 August 2024/Tismana City, Gorj County (45°05′23.8″ N, 22°55′06.2″ E)	GAL-BIF-2024-0819-2
Gb_2	*G. bifida*/*herba*	19 August 2024/Tismana City, Gorj County (45°05′23.8″ N, 22°55′06.2″ E)	GAL-BIF-2024-0819-2
Gb_3	*G. bifida*/*folium*	19 August 2024/Tismana City, Gorj County (45°05′23.8″ N, 22°55′06.2″ E)	GAL-BIF-2024-0819-2
Gs_1	*G. speciosa*/*radix*	19 August 2024/Tismana City, Gorj County (45°05′18.2″ N, 22°55′06.1″ E)	GAL-SPC-2024-0819-2
Gs_2	*G. speciosa*/*herba*	19 August 2024/Tismana City, Gorj County (45°05′18.2″ N, 22°55′06.1″ E)	GAL-SPC-2024-0819-2
Gs_3	*G. speciosa*/*folium*	19 August 2024/Tismana City, Gorj County (45°05′18.2″ N, 22°55′06.1″ E)	GAL-SPC-2024-0819-2
Gt_1	*G. tetrahit*/*radix*	21 July 2024/Lăpuşnicel Village, Caraş Severin County (44°59′17.4″ N, 22°13′50.9″ E)	GAL-TTH-2024-0721-2
Gt_2	*G. tetrahit*/*herba*	21 July 2024/Lăpuşnicel Village, Caraş Severin County (44°59′17.4″ N, 22°13′50.9″ E)	GAL-TTH-2024-0721-2
Gt_3	*G. tetrahit*/*folium*	21 July 2024/Lăpuşnicel Village, Caraş Severin County (44°59′17.4″ N, 22°13′50.9″ E)	GAL-TTH-2024-0721-2

## Data Availability

The original contributions presented in this study are included in the article. Further inquiries can be directed to the corresponding author.

## Supplementary Materials

The following supporting information can be downloaded at: https://www.mdpi.com/article/10.3390/ph18040599/s1, Figure S1: UHPLC/UV (265 and 325 nm) chromatograms of *Galeopsis* spp. samples; Table S1: Results (mean ± SD) of the TPC, TFC, antioxidant (DPPH, ABTS, and FRAP), and AChE inhibitory assays for *Galeopsis* spp. samples; Table S2: Concentrations (μg/g) of phenolic acids quantified in *Galeopsis* spp. samples.

## Abbreviations

The following abbreviations are used in this manuscript:

ABTS	2,2′-Azino-*bis*(3-ethylbenzothiazoline-6-sulfonic acid)
AChE	Acetylcholinesterase
AlCl_3_	Aluminum chloride
ANOVA	Analysis of variance
CI	Confidence interval
DPPH	2,2-Diphenyl-1-picrylhydrazyl
FeCl_3_	Ferric chloride
FeSO_4_·7H_2_O	Ferrous sulfate heptahydrate
FRAP	Ferric-reducing antioxidant power
GAE	Gallic acid equivalents
Gb	*Galeopsis bifida*
Gs	*Galeopsis speciosa*
Gt	*Galeopsis tetrahit*
HCl	Hydrochloric acid
HPTLC	High-performance thin-layer chromatography
IC_50_	Half-maximal inhibitory concentration
LC	Liquid chromatography
*m/z*	Mass-to-charge ratio
MS	Mass spectrometry
MS/MS	Tandem mass spectrometry
NMR	Nuclear magnetic resonance
NP–PEG	Natural products–polyethylene glycol
PDA	Photodiode array
QE	Quercetin equivalents
R_f_	Retention factor
RT	Room temperature
SD	Standard deviation
TFC	Total flavonoid content
TPC	Total phenolic content
TPTZ	2,4,6-*Tris*(2-pyridyl)-1,3,5-triazine
UHPLC	Ultra-high-performance liquid chromatography
UV	Ultraviolet
WWPTFE	Water wettable polytetrafluoroethylene
