# Supplementary material for: Phenolic Acid Investigation and In Vitro Antioxidant and Antiacetylcholinesterase Potentials of Galeopsis spp. (Lamiaceae) from Romanian Flora
Source: Pharmaceuticals (Basel). 2025 Apr 20;18(4):599. doi: 10.3390/ph18040599 (PMC12030362; doi:10.3390/ph18040599)
Supplement: Supplementary file 1 [file pharmaceuticals-18-00599-s001.zip › pharmaceuticals-3559947-supplementary.pdf]

Supplementary Material

Article

Phenolic Acids Investigation, *In Vitro* Antioxidant and Antiacetylcholinesterase Potential of *Galeopsis* spp. (*Lamiaceae*) from Romania Flora

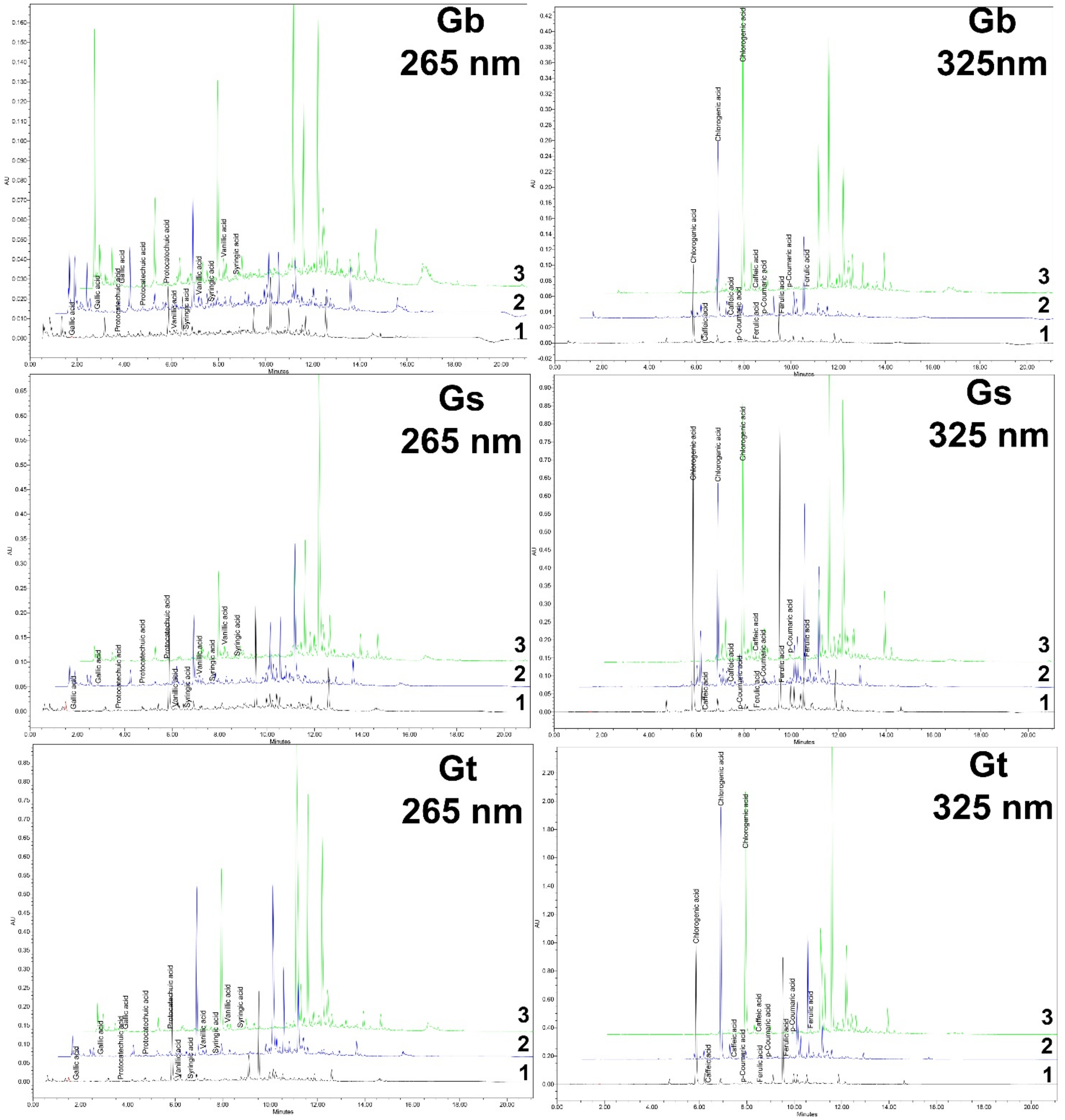

Figure S1. UHPLC/UV (265 and 325 nm) chromatograms of *Galeopsis* samples. 1: Roots; 2: Aerial parts; 3: Leaves; Gb: *G. bifida*; Gs: *G. speciosa*; Gt: *G. tetrahit*. UHPLC: Ultra-high-performance liquid chromatography; UV: Ultraviolet.

Table S1. Results (mean ± SD) of the TPC, TFC, antioxidant (DPPH, ABTS, and FRAP) and AChE inhibitory assays for *Galeopsis* samples.

**Sample**	**TPC (μg GAE/mL)**	**TFC (μg QE/mL)**	**DPPH IC_{50} (mg/mL)**	**ABTS IC_{50} (mg/mL)**	**FRAP (mM)**	**AChE IC_{50} (mg/mL)**
Gb_1	152.674±12.412	22.78±0.663	16.84±0.973	4.772±0.302	10.392±0.401	17.23±0.040
Gb_2	277.596±1.248	57.264±4.604	8.102±0.488	3.2±0.253	11.455±1.125	15.63±1.211
Gb_3	453.423±26.255	207.375±10.324	2.095±0.072	1.124±0.110	18.672±1.256	6.97±0.679
Gs_1	466.504±43.758	97.091±3.063	2.314±0.217	1.416±0.102	19.271±1.432	15.06±0.641
Gs_2	496.698±34.353	273.612±5.159	2.399±0.118	1.665±0.101	19.979±1.751	13.89±1.331
Gs_3	661.952±46.466	343.572±31.855	0.789±0.030	0.594±0.037	24.963±0.682	6.92±0.139
Gt_1	398.702±32.610	128.602±10.446	2.469±0.004	1.631±0.059	19.734±1.935	11.42±0.419
Gt_2	644.947±63.443	336.714±16.499	1.511±0.107	1.086±0.098	25.48±1.156	10.94±0.447
Gt_3	971.203±60.377	568.543±24.174	0.458±0.030	0.328±0.003	37.763±2.517	4.002±0.319

1: Roots; 2: Aerial parts; 3: Leaves; ABTS: 2,2′-Azino-*bis*(3-ethylbenzothiazoline-6-sulfonic acid); AChE: Acetylcholinesterase; DPPH: 2,2-Diphenyl-1-picrylhydrazyl; FRAP: Ferric-reducing antioxidant power; GAE: Gallic acid equivalents; Gb: *G. bifida*; Gs: *G. speciosa*; Gt: *G. tetrahit*; IC_{50}: Half-maximal inhibitory concentration; QE: Quercetin equivalents; SD: Standard deviation; TFC: Total flavonoid content; TPC: Total phenolic content.

Table S2. Concentrations (μg/g) (mean ± SD) of phenolic acids quantified in *Galeopsis* samples.

**Sample**	**Caffeic acid**	**Chlorogenic acid**	***p*-Coumaric acid**	**Ferulic acid**	**Gallic acid**	**Protocatechuic acid**	**Syringic acid**	**Vanillic acid**
Gb_1	2.699± 0.135	609.731± 30.487	15.907± 0.795	11.174± 0.559	0.0± 0.0	55.773± 2.789	32.549± 1.627	30.543± 1.527
Gb_2	9.804± 0.490	1410.156± 70.508	63.725± 3.186	5.756± 0.288	0.051± 0.003	91.224± 4.561	40.539± 2.027	61.394± 3.070
Gb_3	58.23± 2.912	5094.923± 254.746	76.614± 3.831	27.683± 1.384	0.0± 0.0	2.305± 0.115	66.431± 3.322	160.823± 8.041
Gs_1	4.929± 0.246	5043.267± 252.163	63.744± 3.187	39.132± 1.957	0.0± 0.0	66.036± 3.302	36.556± 1.828	35.639± 1.782
Gs_2	70.437± 3.522	3483.651± 174.183	59.879± 2.994	14.199± 0.710	11.128± 0.556	29.652± 1.483	4.763± 0.238	174.401± 8.720
Gs_3	288.87± 14.444	8712.628± 435.631	534.11± 26.706	116.943± 5.847	0.0± 0.0	37.246± 1.862	27.311± 1.366	421.963± 21.098
Gt_1	10.096± 0.505	6271.511± 313.576	64.844± 3.242	12.236± 0.612	2.911± 0.146	23.677± 1.184	29.306± 1.465	48.046± 2.402
Gt_2	47.594± 2.380	11 678.509± 583.925	159.258± 7.963	77.982± 3.899	12.548± 0.627	62.402± 3.120	25.784± 1.289	124.145± 6.207
Gt_3	129.61± 6.481	22 347.907± 1117.395	145.03± 7.252	271.089± 13.554	40.962± 2.048	176.536± 8.827	82.726± 4.136	178.442± 8.922

1: Roots; 2: Aerial parts; 3: Leaves; Gb: *G. bifida*; Gs: *G. speciosa*; Gt: *G. tetrahit*; SD: Standard deviation.
